# The Antitumor Role of Incomptine A in a Breast Cancer Murine Model: Impairment of Hexokinase II Expression and Apoptosis Induction

**DOI:** 10.3390/cells14151192

**Published:** 2025-08-02

**Authors:** Angel Giovanni Arietta-García, Fernando Calzada, Antonio Franco-Vadillo, Irais Monserrat Barrientos-Buendía, Francisco Javier Alarcón-Aguilar, Elihú Bautista, Paola Santana-Sánchez, Israel Ramírez-Sánchez, Rosa María Ordoñez-Razo

**Affiliations:** 1 Posgrado en Biología Experimental, DCBS, Universidad Autónoma Metropolitana-Iztapalapa, Av. Ferrocarril San Rafael Atlixco 186, Col. Leyes de Reforma 1a Secc. Iztapalapa, Ciudad de México C.P. 09310, Mexico; angelo_arietta@hotmail.com; 2 Unidad de Investigación Médica en Genética Humana, Hospital de Pediatría “Dr. Silvestre Frenk Freund”, Centro Médico Nacional Siglo XXI, Instituto Mexicano del Seguro Social, Av. Cuauhtémoc 330, Col. Doctores, Ciudad de México C.P. 06720, Mexico; irais.m3093@gmail.com; 3 Unidad de Investigación Médica en Farmacología, UMAE Hospital de Especialidades, 2° Piso CORSE, Centro Médico Nacional Siglo XXI, Instituto Mexicano del Seguro Socia, Av. Cuauhtémoc 330, Col. Doctores, Ciudad de México C.P. 06720, Mexico; fercalber10@gmail.com; 4 Medicina Hiperbárica Experimental y Farmacología Molecular, SEPI, Escuela Superior de Medicina, Instituto Politécnico Nacional, Salvador Díaz Mirón esq. Plan de San Luis S/N, Casco de Santo Tomas, Ciudad de México C.P. 11340, Mexico; afv2504@gmail.com; 5 Laboratorio de Farmacología, Departamento de Ciencias de la Salud, DCBS, Universidad Autónoma Metropolitana-Iztapalapa, Av. Ferrocarril San Rafael Atlixco 186, Col. Leyes de Reforma 1a Secc. Iztapalapa, Ciudad de México C.P. 09310, Mexico; alaguilar888@gmail.com; 6 CONAHCYT—Consorcio de Investigación, Innovación y Desarrollo para las Zonas Áridas, Instituto Potosino de Investigación Científica y Tecnológica A.C., San Luis Potosí C.P. 78216, Mexico; francisco.bautista@ipicyt.edu.mx; 7 Unidad de Investigación Médica en Inmunología, Hospital de Pediatría “Dr. Silvestre Frenk Freund”, Centro Médico Nacional Siglo XXI, Instituto Mexicano del Seguro Social, Av. Cuauhtémoc 330, Col. Doctores, Ciudad de México C.P. 06720, Mexico; pao-ss@live.com.mx; 8 Sección de Estudios de Posgrado e Investigación, Escuela Superior de Medicina, Instituto Politécnico Nacional, Salvador Díaz Mirón esq. Plan de San Luis S/N, Casco de Santo Tomas, Ciudad de México C.P. 11340, Mexico; i1ramirezsanchez@ucsd.edu

**Keywords:** incomptine A, antitumor activity, breast cancer, hexokinase II, apoptosis

## Abstract

Breast cancer (BC) is the most common type of cancer in women worldwide. Hexokinase II (HKII) overexpression is associated with the proliferation and survival of tumor cells, as it inhibits apoptosis. Incomptine A (IA) is cytotoxic to breast cancer cells, likely due to a decrease in the expression of HKII. This study evaluated the antitumor activity of IA in an in vivo mouse model of BC. A model was generated from 4T1 cells and grouped tumor-bearing animals according to treatment: in IA or doxorubicin (DOXO), or untreated (UT). Comparing the body weight and tumor size between groups, tumors were analyzed using histopathological, Western blot, flow cytometry, and mitochondrial activity assays. Tumors IA-treated showed a reduction in size, weight, and number of tumor cells; the expression of HKII and Bcl-2 decreased, while that of Caspase-3 increased. IA treatment increased apoptosis and reduced mitochondrial activity in tumor cells. This data showed that IA has an impact on tumor cells by reducing tumor volume and size, increasing cell apoptosis, and decreasing mitochondrial activity, all of which could be attributed to reduced HKII expression. Therefore, IA may be a promising compound that requires further studies to elucidate its mechanism of action and analyze its possible future use in BC.

## 1. Introduction

Breast cancer (BC) has the highest incidence and mortality in women worldwide and represents approximately 23.8% of reported cases and 15.4% of deaths caused by cancer [1]. In México, only in 2022, 23,790 new cases and 7888 deaths from BC were reported, making BC a significant public health problem [2].

BC can be classified molecularly into four categories, HER-2+ (human epidermal growth factor receptor 2), luminal A, luminal B, and triple-negative, which differ in factors such as incidence, survival, and treatment [3]. Because of this, specific medications are required for each subtype; however, it has been reported that tumor cells can generate resistance to drugs and can also present adverse effects that impact the patients [4,5]. Because of this, it is vitally essential to search for new treatments that are more effective and selective against cancer cells. A necessary source for the obtention of new anticancer agents is the natural products obtained from medicinal plants, which have contributed significantly to pharmacotherapy [6].

Sesquiterpene lactones are a subclass of terpenoids, with structural diversity and confirmed antitumor activity in different types of cancer [7]. Incomptine A (IA) is a sesquiterpene lactone that exhibits cytotoxic and antitumor activities in non-Hodgkin’s lymphoma cells [8,9] and in BC cell lines [10]. In BC, IA has shown a similar cytotoxic activity among luminal A, HER-2(+), and triple-negative subtypes as well as greater selectivity towards cancer cells, since much less damage was observed in non-tumorigenic cells [10], which suggests that IA could be a compound that meets the characteristics of universality and selectivity necessary to be considered in studies aimed at analyzing its possible use as a phytopharmaceutical for BC.

Hexokinase II (HKII) is a glycolytic enzyme that is often found to be overexpressed in various types of cancer. In BC, this increased expression is correlated with more advanced histological grades, increased proliferative activity, increased risk of recurrence, and adverse clinical outcomes [11,12]. HKII also participates in regulating apoptotic signaling by inhibiting cell death by binding to the voltage-dependent anion channel 1 (VDAC1) located in the mitochondrial outer membrane (MOM). HKII competes with pro-apoptotic molecules, such as BAX and BAK, to bind to VDAC1, thereby inhibiting apoptosis [13,14,15]. Based on this, HKII is considered an important therapeutic target in cancer.

A previous report demonstrated that IA treatment in different BC cell lines reduces HKII expression but not the expression of other glycolytic enzymes [10]. The same report suggested that the HKII reduction could be due to the strong interaction between IA and HKII observed in molecular docking analyses [10]. The comparison of the IA-HKII union site to the union site for 2-Deoxy-D-glucose (2-DG), an HKII inhibitor, revealed that IA and 2-DG share a similar binding site for HKII [10]. Based on this, it is proposed that the cytotoxic mechanism of IA involves its binding to HKII, thereby blocking HKII activity and releasing VDAC1, which consequently induces apoptosis in these cancer cells [10]. However, it is necessary to demonstrate that the reported IA activity in BC cell lines is the same in an in vivo tumor model. Therefore, this study aimed to examine the antitumor activity of IA in a murine model of BC and its effect on the expression of HKII and apoptosis-related proteins.

## 2. Materials and Methods

### 2.1. Incomptine A

Dr. Elihú Bautista provided the IA. The IA was purified from the leaves of *Decachaeta incompta* according to the reported methodology by Bautista et al. (2012) [16]. IA identification was performed using nuclear magnetic resonance spectroscopy with an authentic sample of 99% purity.

### 2.2. Cell Culture

The 4T1 cell line (cat CRL-2539) was acquired from the American Type Culture Collection (ATCC, Manassas, VA, USA). The 4T1 cell line was cultivated in RPMI-1640 medium (Thermo Fisher Scientific, Waltham, MA, USA) with 100 µg/mL of penicillin/streptomycin (Thermo Fisher Scientific, Waltham, MA, USA) supplemented with 10% fetal bovine serum (Thermo Fisher Scientific, Waltham, MA, USA). The cells were incubated and maintained in a humidified atmosphere at 37 °C and 5% CO_2_ until they were 80% confluent.

### 2.3. Laboratory Animals

To generate the murine model, virgin female Balb/c mice weighing 22 ± 2 g were used. The mice were obtained from the bioterium of the Centro Médico Nacional SXXI del Instituto Mexicano del Seguro Social (IMSS). Clean and sterile polyvinyl cages were maintained during the investigation. The mice were manipulated according to the specifications of the Norma Oficial Mexicana NOM-062-ZOO-1999 [17] for the production, care, and use of laboratory animals. All investigations were performed with the endorsement of the Comité de Ética and the Comité Científico de Investigación en Salud del Instituto Mexicano del Seguro Social, Siglo XXI.

### 2.4. Acute Oral Toxicity

An evaluation of the acute oral toxicity of IA and the control drug doxorubicin was performed at lethal dose 50 (LD_50_), which is the dose at which 50% of the treated animals can die, and according to guideline 423 described by the OECD for the evaluation of acute oral toxicity of chemical substances [18]. Eighteen mice of the BALB/c strain (females) weighing 20 ± 2 g were used for each group. The mice were fasted for 4 h before a single administration of IA and DOXO at doses of 100, 150, and 200 mg/kg, administered oral-intragastrically. The animals were observed for 4 h after the administration of the treatments to verify any abnormalities such as diarrhea, convulsions, spasms, vomiting, coma, or mortality. The observation occurred once a day for 15 days, and the mice were then sacrificed to perform an autopsy and rule out any macroscopic damage to the organs. Subsequently, the LD_50_ values for IA and DOXO were determined using linear regression in the GraphPad Prism version 8 program (GraphPad Software, San Diego, CA, USA). Finally, their toxicity category, IA and DOXO, was determined according to the OECD categories: Toxicity Category I (highly toxic), Toxicity Category II (moderately toxic), Toxicity Category III (slightly toxic), and Toxicity Category IV (practically non-toxic) [18].

### 2.5. Murine Model of Breast Cancer

A murine model of BC was created according to Pulaski’s protocol [19]. 4T1 cells (1 × 10^5^ cells) in 100 µL of culture medium were inoculated subcutaneously around the left lower mammary gland of the mice. Subsequently, tumor formation was verified by palpation 7 days after inoculation. Once the tumor was implanted, the mice were randomly assigned to groups.

### 2.6. Antitumor Activity

The mice were assigned to five groups (n = 6): a UT group; a DOXO treatment group (80 mg/kg); and three IA treatment groups (0.07, 0.7, and 2.5 mg/kg). The doses of IA used in this work were selected based on the cytotoxic concentration 50 (CC_50_) reported by Arietta et al. (2023) [10]. The CC_50_ of 7 µg/mL of the cell lines was multiplied at 10X, 100X, and 350X according to the methods of Schlie-Guzman [20]. The final doses administered to the mice were 0.07, 0.7, and 2.5 mg/kg. All treatments were administered orally, one dose daily for 8 days. Day 1 of the treatment was day 7 post-inoculation, and day 8 was day 14 after cell inoculation. The tumors were measured with a vernier beginning on day 12 after cell inoculation and then every 2 days, until day 28, which was the day the animals were euthanized. Tumor volume (VT) was calculated with the formula VT = A × B2 × 0.5, where A is the longest diameter of the tumor and B is the shortest diameter of the tumor. The weight and survival of the animals were recorded weekly for 4 weeks. The antitumor activity was determined by comparing the average weight of the mammary tumor growth in the animals treated with IA against those without treatment. The effective dose 50 (ED50) of IA was then calculated by linear interpolation obtained from the data on the percentage of decrease in tumor growth. The weight and survival of the animals were recorded weekly for 4 weeks. The antitumor activity was determined by comparing the average weight of the mammary tumor growth in the animals treated with IA against those without treatment. Finally, the effective dose 50 (ED_50_) of IA was calculated by linear interpolation obtained from the data on the percentage of decrease in tumor growth.

### 2.7. Toxicity and Safety Profile of IA in the Murine BC Model

The blood obtained was incubated for 30 min at room temperature and then centrifuged at 3000 RPM for 15 min at 4 °C to obtain total serum, which was used to measure the levels of glutamic-pyruvic transaminase (SGPT) and glutamic-pyruvic transaminase oxalacetic acid (SGOT) using the SPOTCHEM EZ SP-4430 automated biochemistry analyzer (Arkray Global Business Inc, Miami, FL, USA).

### 2.8. Histopathological Analysis

The tumors were divided into four sections, and then, one section was fixed in paraffin. The latter was then sectioned into 7 µm sections using a microtome and placed on a slide, where it was subjected to hematoxylin–eosin (H&E) staining. An analysis was performed using a high-resolution microscope connected to a digital camera (Exview HAD; Sony México, Ciudad de México, México) and a standard computer (IBM computer system, Ciudad de México, México).

### 2.9. Western Blot

A portion of the breast tumor samples was used for protein extraction. Equal amounts of protein for the UT, IA-treated, and DOXO-treated groups were separated by SDS-PAGE; transferred to a PVDF polyvinylidene difluoride membrane (Merck, Rahway, NJ, USA. IPVH00010); blocked with 5% skim milk; and incubated overnight with primary antibodies against hexokinase II (HKII) (1:2500, Santa Cruz Biotechnology, Dallas, TX, USA. sc-130358), aldolase A (ALDOA) (1:2500, Santa Cruz Biotechnology, Dallas, TX, USA. sc-390733), lactate dehydrogenase A (1:2500, Santa Cruz Biotechnology, Dallas, TX, USA. sc-133123), caspase 3 (1:2500, Santa Cruz Biotechnology, TX, USA. sc-56053), and Bcl-2 (1:2500, Santa Cruz Biotechnology, Dallas, TX, USA. sc-7382). Horseradish peroxidase (HRP)-conjugated donkey anti-mouse antibody (1:2500, Abcam, Cambridge, UK, ab6820) was used for secondary detection, and the reaction was visualized using a chemiluminescent reagent (Thermo Fisher Scientific, Waltham, MA, USA). A densitometry analysis was quantified using ImageJ software version 1.53 (National Institutes of Health, Bethesda, MD, USA). The relative expression of the analyzed proteins between the untreated and IA-treated samples was calculated after normalizing to the intensity of the β-actin (1:5000, Santa Cruz Biotechnology, Dallas, TX, USA. sc-47778) band for each sample.

### 2.10. Annexin V/Ghost Red Staining

A total of 20 mg of tumoral tissue from the DOXO 80 mg/kg and IA 2.5 mg/kg groups was incubated for one hour with 400 U/mL collagenase I (Thermo Fisher Scientific, Waltham, MA, USA, 100017) at 37 °C. Subsequently, the enzymatic reaction was stopped with 0.1 M EGTA and filtered through a 70 μm cell strainer (Thermo Fisher Scientific, Waltham, MA, USA, 352350) to obtain a cell suspension containing the cancer cells. The 4T1 tumor cancer cells were then sorted using the CD44 Monoclonal Antibody (IM7), conjugated to PE-Cyanine5 (Thermo Fisher Scientific, Waltham, MA, USA, 15-0441-82). Cell apoptosis was analyzed using the FITC Annexin V Apoptosis Detection Kit (BioLegend, San Diego, CA, USA, 640922), and cell viability was assessed with Ghost Dye Red 780 Viability Dye (Tonbo Biosciences, San Diego, CA, USA, 13-0865). The samples were processed, and 50,000 events were acquired on a MACSQuant Analyzer 10 flow cytometer (Miltenyi Biotec B.V. & Co. KG, Bergisch Gladbach, GER). Finally, the data were analyzed with FlowJo 10 software (BD Biosciences, Ashland, OR, USA).

### 2.11. Mitochondrial Respiratory Complex I Activity Assay

For the analysis of mitochondrial activity, 100 mg of tissue from each tumor was used. The mitochondria were purified using the MitoCheck Mitochondrial (Tissue) Isolation Kit (Cayman Chemicals, Ann Arbor, MI, USA) following the manufacturer’s instructions. Then, 20 μg of mitochondrial protein was used to determine the mitochondrial respiratory complex I activity with the MitoCheck Complex I activity Assay Kit (Cayman Chemicals, Ann Arbor, MI, USA), and the absorbance of each sample was measured for 30 s intervals for 15 min at 25 °C and at 340 nm in a plate reader. Complex I activity resulted in a decrease in absorbance per minute. Standard error (SE) values were obtained from three sets of biological replicates.

### 2.12. Statistical Analysis

The results are expressed as the mean ± SE of six measurements and were statistically analyzed using GraphPad Prism version 8 (GraphPad Software, San Diego, CA, USA), which performed a one-way ANOVA. Multiple comparison tests were performed with Dunnett’s test with a *p*-value less than 0.05 to establish significant differences between study groups.

## 3. Results

### 3.1. Acute Oral Toxicity of Incomptine A

The acute oral toxicity of IA and DOXO was evaluated in agreement with OECD guidelines 423 for the use of natural products in human consumption [18]. The LD_50_ for IA was 149.0 ± 0.50 mg/kg, and for DOXO, it was 100 ± 0.50 mg/kg. Therefore, based on the OECD, the LD_50_ values for IA and DOXO fall within category 3 (LD_50_ = 50–300 mg/kg), indicating that both can be considered slightly toxic. Hence, IA can be used in animal models without problems, as its risk is minimal.

### 3.2. Antitumor Activity of Incomptine A

**Figure 1:**
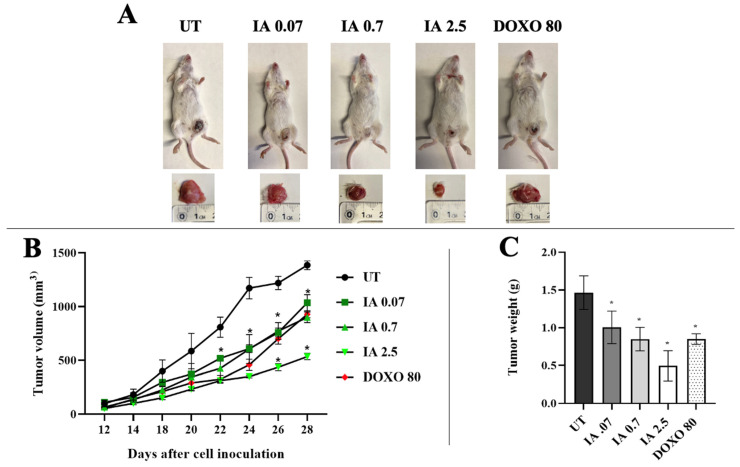
The antitumor effect of IA treatment on the in vivo model of BC. (**A**) Treated mice with IA (0.07, 0.7, and 2.5 mg/kg); the animals had extirpated tumors after treatment. (**B**) Tumor volumes were measured and calculated daily. (**C**) The tumor mass was weighted. The data are expressed as mean ± SEM (n = 6). * *p* < 0.05 vs. untreated group. Abbreviations: IA (incomptine A), DOXO (doxorubicin), UT (untreated).

The antitumor effect of IA was evaluated in vivo in an orthotopic murine tumor model. Representative tumor sizes for each group are shown in Figure 1A. The average tumor volume in the UT group was 1385 mm^3^ (Figure 1B). In the IA treatment group, the tumor volume was less in the three doses (0.07, 0.7, and 2.5 mg/kg); the mean tumor volume decreased to 1035, 900, and 535 mm^3^, respectively. In contrast, the average tumor volume for treatment with the control drug DOXO (80 mg/kg) was 928 mm^3^. Therefore, IA treatment reduced tumor volume in a dose-dependent manner by up to 61% compared with the UT group and by 42% compared with the DOXO group (Figure 1B).

The excised tumors were weighed, and the reduction in tumor growth was analyzed (Figure 1C). The IA treatment groups showed a greater decrease in tumor growth in the three doses analyzed compared to the UT group. The IA at 0.07 mg/kg reduced tumor growth by approximately 31%, while the 0.7 mg/kg dose reached a 42% reduction. The highest dose of IA tested (2.5 mg/kg) produced the most pronounced effect, with a 66% reduction in tumor growth. In contrast, the treatment with DOXO (80 mg/kg) resulted in a 42% reduction in tumor growth relative to the UT group (Figure 1C).

The ED_50_ for both IA and DOXO was determined from the data on the percentage decrease in tumor growth. The ED_50_ for IA was 2.00 ± 0.12 mg/kg, and for DOXO, it was 86.2 ± 5.5 mg/kg. Therefore, IA can reduce tumor size with a much lower dose than DOXO. The therapeutic index (TI) was calculated with the LD_50_/ED_50_ ratio. TI is used to assess whether a safe compound is safe. The TI value for IA was 74.5, and for DOXO, it was 1.16. This result indicates that IA is safer and less toxic than DOXO, since a compound is considered safe when its TI is high [21].

### 3.3. Histopathology Analysis of Tumors

**Figure 2:**
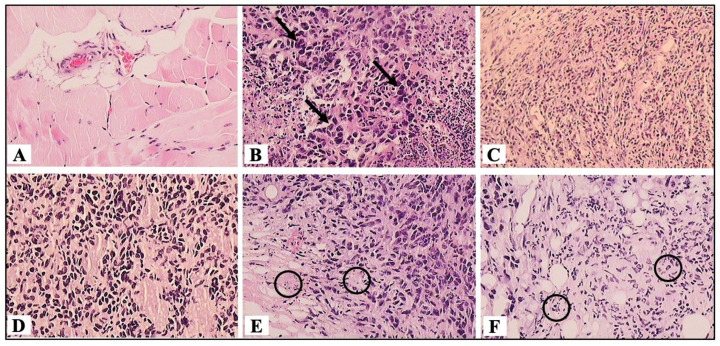
Representative photomicrographs with hematoxylin–eosin (H&E) staining of mouse mammary tumors induced by 4T1 cells observed at 20×. (**A**) The healthy breast tissue of mice; (**B**) untreated mouse mammary tumor; (**C**) mouse mammary tumor treated with DOXO; (**D**) mouse mammary tumor treated with IA 0.07 mg/kg, (**E**) 0.7 mg/kg, and (**F**) 2.5 mg/kg. The data were generated from six fields per slide, with four slides analyzed from each tumor, and three tumors were examined from each group. (n = 6). The black arrows indicate viable tumor cells, and the black circles indicate apoptotic bodies.

To analyze the histopathological changes in the IA-treated tumors, hematoxylin–eosin staining was used. The UT tumors shown exhibited structural loss and were mostly viable, with tumor cells displaying prominent pleomorphic, vesicular, and hyperchromatic nuclei (Figure 2B, black arrows). A pattern of central necrosis was accompanied by peripheral lymphocytic infiltration and fibrosis of the surrounding connective tissue. In comparison, the IA-treated mice showed greater preservation of the surrounding connective tissue as well as a significant decrease in the number of viable tumor cells and an increase in apoptotic cells (Figure 2D–F), and the presence of apoptotic bodies (black circles, Figure 2E,F). On the other hand, DOXO also showed a decrease in cellularity (Figure 2C), albeit to a lesser extent than the IA-treated group. These observations indicate that IA is decreasing the number of cancer cells by inducing cellular apoptosis.

### 3.4. Toxicity of Incomptine A Treatment in an In Vivo Mouse Model

**Figure 3:**
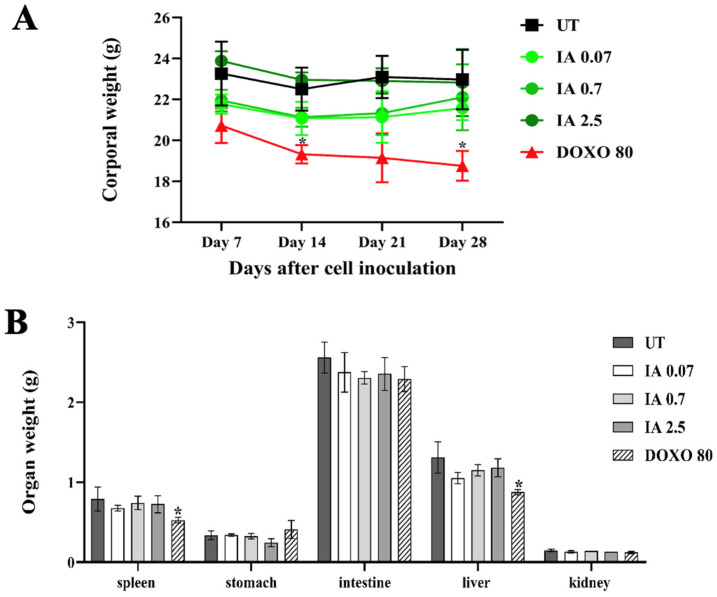
The effect of IA treatment on the corporal and organ weight of mice at the end of the treatment. (**A**) The corporal weight of the mice was measured weekly from days 7 to 28 after cell inoculation. (**B**) The weight of the organs obtained at the end of the experiment. All data were obtained from three independent experiments and expressed as the mean value ± SEM (n = 6). * *p* < 0.05 vs. untreated group. Abbreviations: IA (incomptine A), DOXO (doxorubicin), UT (untreated).

The systemic toxicity of IA was evaluated in the in vivo model. In all groups, body weight, organ weight, and hepatotoxicity were measured (Figure 3). None of the mice treated with IA showed changes in their body weight over time, like the UT group. Only the DOXO group showed a significant decrease in body weight (Figure 3A). Gross necropsy did not show any sign of toxicity, and the weights of the spleen, stomach, intestine, liver, and kidney were not different between the IA and the UT groups. However, there was a minor weight difference between the spleen and liver in the DOXO group (Figure 3B), which could indicate toxicity in these organs.

To analyze the hepatotoxicity, the plasma levels of indicators of liver cell injury, such as glutamic-oxaloacetic transaminase (SGOT) and serum glutamic-pyruvic transaminase (SGPT), were measured (Table 1). In the plasma of all the IA-treated mice, there were no changes in the SGOT and SGPT profiles, with these values being very similar to those obtained in healthy mice without tumors. In contrast, the UT mice showed higher values in both enzymes. On the other hand, in the DOXO-treated mice, only SGOT levels increased significantly. Therefore, these data indicate that IA has an acceptable safety profile.

### 3.5. Treatment with Incomptine A Decreases HKII Expression in Mammary Tumors of Mice

**Figure 4:**
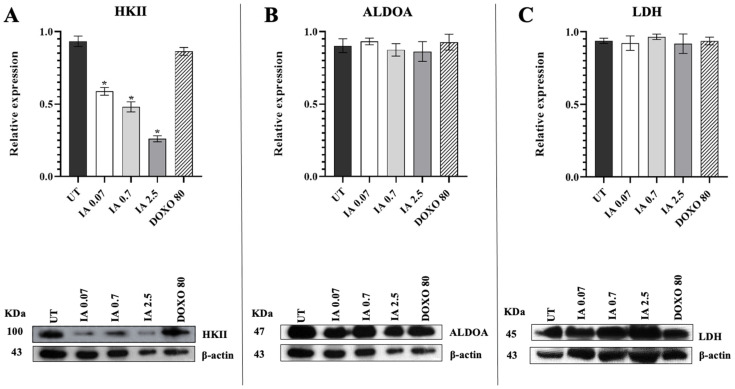
Expression of glycolytic enzymes in tumors of the untreated group and the groups treated with IA (0.07, 0.7, and 2.5 mg/kg) and DOXO (80 mg/kg). (**A**) Relative expressions of HKII, (**B**) ALDOA, and (**C**) LDH. Data are represented as the mean ± SEM of 3 independent experiments. * *p* < 0.05 vs. untreated group. Abbreviations: IA (incomptine A), DOXO (doxorubicin), UT (untreated), HKII (hexokinase II), ALDOA (aldolase A), LDH (lactate dehydrogenase).

To study the effect of IA on some of the enzymes of the glycolytic pathway in mouse tumors, the expression of HKII, aldolase A (ALDOA), and lactate dehydrogenase (LDH) was analyzed in a Western blot (Figure 4). The HKII expression in the tumors treated with the three doses of IA (0.07, 0.7, and 2.5 mg/kg) decreased significantly (65%, 80%, and 90%, respectively) in comparison with tumors from the UT group (Figure 4A). Regarding the expression of ALDOA and LDH, no differences were found to be significant in its expression in tumors IA-treated compared to those not treated (Figure 4B). The mice treated with DOXO did not present modification significance in the expression of any of the other enzymes analyzed (Figure 4B,C). These data suggest that the antitumor effect of IA is perhaps directly related to HKII.

**Table 1:** Plasma levels of glutamic-oxaloacetic transaminase (SGOT) and serum glutamic-pyruvic transaminase (SGPT) in healthy tumor-free mice, untreated mammary tumor mice, and mice treated with incomptine A and doxorubicin.

Mice	Treatment (mg/kg)	SGOT	SGPT
Healthy tumor-free	untreated	250.2 ± 11	49 ± 5.80
Mammary tumor	untreated	970.5 ± 29 *	445 ± 17 *
	0.07	213.6 ± 10	41.6 ± 6
IA	0.70	244.3 ± 4	43.3 ± 9
	2.50	264.6 ± 4	44.6 ± 6
DOXO	80.00	478.6 ± 4 *	54.6 ± 9

Data expressed as the mean ± SEM (n = 6). * *p* < 0.05 vs. healthy tumor-free mouse. IA = incomptine A; DOXO = doxorubicin.

### 3.6. Incomptine A Induces Apoptosis in Breast Cancer Tumor Cells

**Figure 5:**
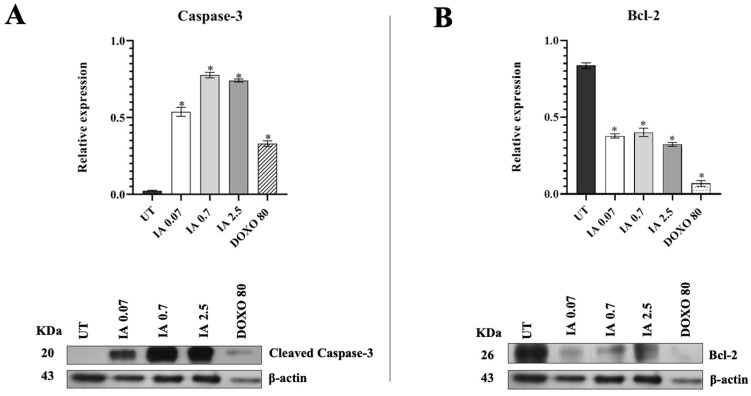
Expression of (**A**) caspase 3 and (**B**) Bcl-2 in tumors of an untreated group and groups treated with IA (0.07, 0.7, and 2.5 mg/kg) and DOXO (80 mg/kg). The data are represented as the mean ± SEM of 3 independent experiments. * *p* < 0.05 vs. untreated group. Abbreviations: IA (incomptine A), DOXO (doxorubicin), UT (untreated).

Because there were no modifications in the other glycolytic enzymes and because HKII also participates in the inhibition of apoptosis, we analyzed the effect of IA on apoptosis in the tumor cells. The anti-apoptotic protein Bcl-2 and caspase-3 were quantified by Western blot densitometry in the tumors of mice treated with IA (0.07, 0.7, and 2.5 mg/kg) and DOXO (80 mg/kg), compared to the tumors of UT mice (Figure 5). These assays showed that the expression of caspase-3 increased significantly in mice receiving the three doses of IA (0.07, 0.7, and 2.5 mg/kg). In the group treated with DOXO (80 mg/kg), a significant increase in caspase-3 was also observed compared to the UT tumors (Figure 5A); however, this increase was much smaller than that observed with IA. On the other hand, the expression of the anti-apoptotic protein Bcl-2 decreased significantly in mice treated with the three doses of IA and DOXO compared to the UT tumors (Figure 5B).

**Figure 6:**
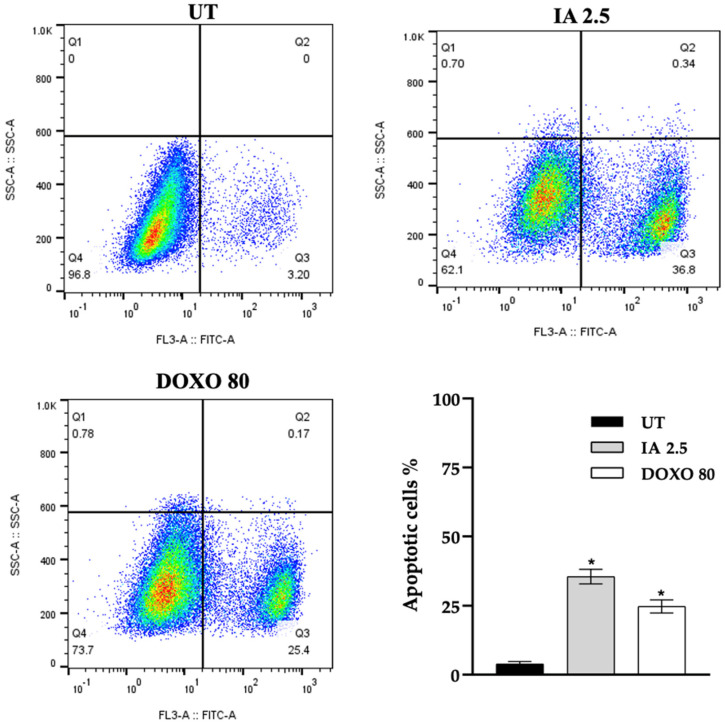
Induced apoptosis in tumor cells after treatment with IA and DOXO. Representative dot plots of apoptosis for IA (2.5 mg/kg) and DOXO (80 mg/kg) treatments and untreated tumor cells. The bar graph compares the percentage of apoptosis between the IA and DOXO treatments with the UT group. The data are expressed as mean ± SEM, n = 3; * *p* < 0.05 versus the untreated group. Abbreviations: IA (incomptine A), DOXO (doxorubicin), and UT (untreated).

The results of flow cytometry showed that IA (2.5 mg/kg) and DOXO (80 mg/kg) treatments increased the proportion of apoptotic cells in the tumors by 33.6% and 22.2%, respectively, compared with the UT group (Figure 6). These results suggest that IA may be associated with the increase in apoptosis in mouse mammary tumors.

### 3.7. Incomptine A Decreases the Mitochondrial Activity of Breast Cancer Tumor Cells

**Figure 7:**
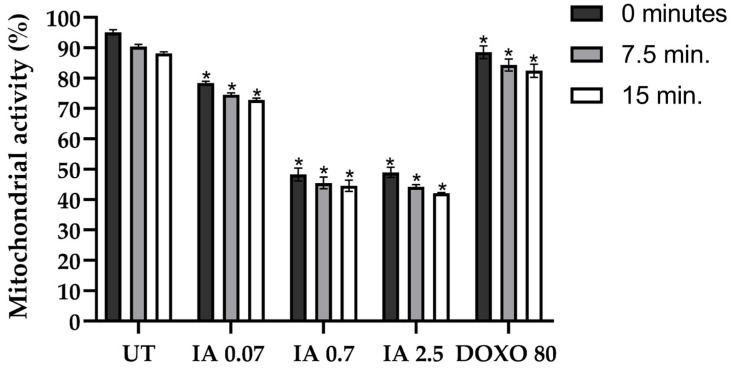
The mitochondrial activity of breast tumor cells after treatment with IA (0.07, 0.7, and 2.5 mg/kg) or DOXO (80 mg/kg) for 15 min. Normalized activities are depicted in untreated tumor cells and represented data as the mean ± SEM of 3 independent experiments. * *p* < 0.05 vs. untreated group. Abbreviations: IA (incomptine A), UT (untreated).

Due to the fundamental role of mitochondrial activity in cell death, we analyzed the modifications in mitochondrial function in mouse mammary tumors treated with 0.07, 0.7, and 2.5 mg/kg of IA. Mitochondrial activity in all IA treatment tumors decreased to 0, 7.5, and 15 min compared to mice without treatment (Figure 7). Mitochondrial activity decreased by 16.35% to 0.07 mg/kg, 46.84% with 0.7 mg/kg, and 46.16% to IA 2.5 mg/kg at 0 min. At 7.5 min, the decrease was 15.84%, 44.9%, and 46.16% with IA 0.07, 0.7, and 2.5 mg/kg, respectively. Finally, at 15 min, a decrease of 15.3% was observed with IA 0.07 mg/kg, 43.63% with IA 0.7 mg/kg, and 45.98% with IA 2.5 mg/kg (Figure 7). On the other hand, in tumors treated with DOXO at 80 mg/kg, mitochondrial activity decreased by 6.57% at 0 min, 6.04% at 7.5 min, and 5.67% at 15 min. The decrease in mitochondrial activity observed in tumor cells IA-treated is consistent with the hypothesis that IA could be participating in the induction of apoptosis.

## 4. Discussion

BC is one of the leading causes of death worldwide. For several years, efforts have been made to develop more effective treatments with universal characteristics that can be used in all subtypes of this cancer (luminal A, luminal B, HER2+, and triple-negative) and with fewer side effects; however, this goal has not yet been achieved. In this context, we studied the effects of the naturally occurring compound incomptine A in a mouse model of BC.

Due to the first-time use of IA as a treatment in an in vivo model for BC, several doses were used to obtain the ED_50_. The IA inhibitory activity on the tumor was observed from the initial dose but increased to more than 50% with a dose of 2.5 mg/kg (ED_50_ = 2.006 ± 0.12 mg/kg). The comparison with DOXO (ED_50_ = 86.2 ± 5.5) showed that to reduce tumor growth, a much lower dose of IA is required; therefore, IA has a greater antitumor effect and could be more effective in reducing tumor mass than DOXO. These results are consistent with those previously reported in a murine lymphoma model, where IA also reduced tumor size with an ED_50_ of 2.4 ± 0.10 mg/kg [9]. Similarly, other sesquiterpene lactones such as parthenolide and costunolide have shown antitumor effects in an in vivo model at doses lower than 10 mg/kg [22,23].

Additionally, IA can be an appropriate compound for use in BC, as it is safer and less toxic due to its TI (74.5) value, which is higher compared to the TI (1.16) for DOXO. A low TI value, as in DOXO, implies that the dosed drug should be used with caution and the person receiving it should be closely monitored for any signs of toxicity [21]. The drug DOXO is an anthracycline, which is interspersed in the DNA of the cell, inhibiting its synthesis and causing cell death. This mechanism is not specific and affects both cancerous and normal cells, leading to various side effects and adverse effects in patients [24], such as weight loss due to skeletal muscle atrophy [25], splenic contraction [26], and hepatotoxicity [27]. In this work, we observed that DOXO-treated mice exhibited changes in body weight and an increase in liver damage markers compared to UT and healthy mice.

In contrast, IA-treated mice maintained their body weight throughout the study, showed no organ damage, and exhibited normal liver damage markers. These results reinforce the evidence that IA has a significantly larger safety margin and less toxicity compared to DOXO. Therefore, IA, in addition to exhibiting antitumor activity at low doses, is also considered safe for use.

BC is a heterogeneous disease, in which each subtype has a different capacity for proliferation and metastasis; however, all subtypes are characterized by metabolic reprogramming, specifically a high glycolytic metabolism and low mitochondrial respiration, which promotes the resistance and survival of cancer cells [28,29]. Alterations in the activity and expression level of several metabolic enzymes, such as HKII, are closely related to cancer progression [30]. HKII is overexpressed in all subtypes of invasive breast carcinomas [31], so it has been the subject of several investigations on its role in BC. These studies have shown that HKII is involved in the acceleration of glycolysis [32] and in the inhibition of apoptosis [13]. Regarding apoptosis in cancer cells, HKII acts as an anti-apoptotic molecule, competing directly with pro-apoptotic molecules such as BAX and BAK, and consequently prevents the release of cytochrome C (Cyt C) and inhibits apoptosis [33].

A previous report showed that IA treatment in BC cell lines decreased HKII expression but did not affect the expression of other glycolytic enzymes such as ALDOA and LDH, suggesting that the reduction in HKII expression would mainly affect their participation in the inhibition of apoptosis due to the likely direct binding of IA with HKII observed in the molecular docking analysis [10]. The binding strength of IA with HKII was greater than that of 2-DG, a previously described inhibitor of HKII [10]. In this work, we evaluated whether IA treatment in a mouse model of BC exhibited the same effect. We also observed that, like in the in vitro model, in the in vivo mice model, IA treatment did not significantly modify the expression of glycolytic enzymes ALDOA and LDH, but the expression of HKII decreased considerably, which supports the hypothesis that HKII could be the target of IA, which acts like an HKII inhibitor [10]. Although the role of 2-DG as an HKII inhibitor is well established, having been used as an antitumor agent in clinical trials [34], it appears to bind to the HKII binding site similarly to IA [10]. In this work, it was not considered as a control molecule instead of DOXO because one of its main limitations is its low efficacy as monotherapy, since it requires concomitant administration with other chemotherapy regimens or at high doses to achieve significant therapeutic effects [35,36,37], which could complicate the analysis of the results.

We also evaluated the induction of apoptosis in the in vivo model caused by IA treatment. Firstly, in the histological sections of the tumors treated with IA, cells were observed to undergo a straightforward apoptotic process, as well as the presence of apoptotic bodies, which could explain the decrease in the number of cancer cells and, consequently, the reduction in tumor size. Subsequently, in the analysis of protein expression related to apoptosis, a clear decrease in the anti-apoptotic protein Bcl-2 and an elevation in the expression of caspase 3 were observed in the tumors treated with IA compared to the UT group. In addition, flow cytometry analyses were performed to determine the presence of apoptosis in the tumor cells obtained from the tumors treated with IA 2.5 mg/kg, and it was observed that the percentage of tumor cells in apoptosis increased when the tumor was treated (33.6%) compared to the cells of the UT tumors (3.2%). Overall, these three analyses evidenced the greater presence of apoptosis in the tumors of mice treated with IA; hence, we could suggest that the induction of apoptosis in these cells is a consequence of the union of IA with HKII, which releases VDAC1 and thus induces apoptosis. However, more studies are necessary to verify this hypothesis.

Due to the observed increase in apoptosis in IA-treated tumor cells and the crucial role mitochondria play in this process, we analyzed mitochondrial activity in the breast tumors of the in vivo model and quantified the mitochondrial complex I activity over 15 min, with a clear decrease in activity observed in tumors treated with IA, particularly at the lowest dose (0.07 mg/kg), compared to the UT group. These results provide evidence of the potential interaction between IA and HKII, as this union would prevent the formation of a binding complex between HKII and VDAC1, thereby altering the mitochondrial membrane potential and potentially leading to mitochondrial dysfunction, as well as an increase in reactive oxygen species (ROS). Other sesquiterpene lactones, such as Parthenolide, Cynaropicrin, and Deoxyelephantopin, have been shown to induce oxidative stress and mitochondrial dysfunction in various types of cancer. It has been observed that these compounds increase the production of ROS within cancer cells. Moreover, they affect mitochondrial membrane potential. These effects compromise the viability of cancer cells by interfering with critical cellular functions and potentially inducing apoptosis [38,39,40]. Regarding IA, it has been reported that it generates ROS in non-Hodgkin’s lymphoma cells [8], but this has not been verified in BC; therefore, it would be ideal to analyze it further.

**Figure 8:**
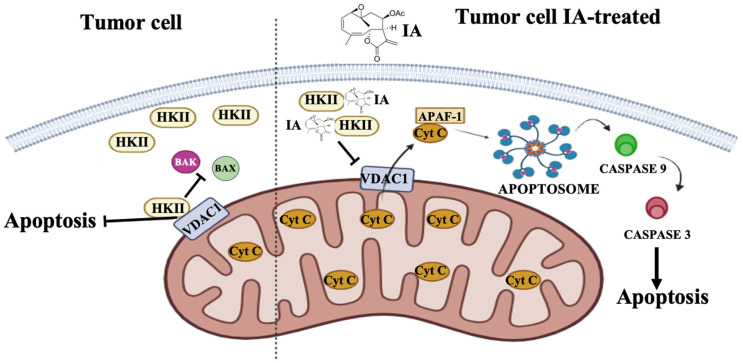
Proposed mechanism of action for IA in BC tumors. In cancer cells, HKII is overexpressed and binds to VDAC1, preventing apoptosis. When mice are administered IA, it binds to HKII, preventing the binding of HKII to VDAC1 and leaving the pore free, releasing pro-apoptotic molecules such as Cyt C, as well as increasing the presence of pro-apoptotic molecules such as caspase 3, with which apoptosis can be induced, and therefore decreasing tumor volume and weight. Abbreviations: IA (incomptine A), HKII (hexokinase II), VDAC1 (voltage-dependent anion channel 1), APAF-1 (apoptosis protease-activating factor-1), Cyt C (cytochrome C).

In a previous report, a possible mechanism of action of IA in BC cell lines involves IA binding to HKII, which, in turn, induces apoptosis. Molecular docking of IA against HKII suggested strong binding to HKII [10]. When IA binds to HKII, it traps it and prevents it from associating with VDAC1 in the MOM, resulting in the induction of apoptosis. In this in vivo model, we observed results like those from the in vitro model [10]; therefore, the previously proposed mechanism is consistent in both models. We suggest that IA binding to HKII causes a reduction in its expression, leading to the induction of apoptosis, which in turn can result in a decrease in the size and weight of the BC tumors in mice (Figure 8). With this data, we propose that IA could be considered a candidate for more in-depth studies aimed at analyzing whether it can be used as a phytomedicine.

## 5. Conclusions

IA demonstrated significant antitumor activity in a murine model of BC by altering HKII expression, which induced apoptosis and mitochondrial dysfunction in tumor cells, thereby reducing the tumor size and weight. Therefore, IA may be a promising compound for further investigation into its potential use in breast cancer.

## Data Availability

All data are available on request to romaorr@yahoo.com.mx.

## Abbreviations

The following abbreviations are used in this manuscript:

BC	Breast cancer
HER-2+	Human epidermal growth factor receptor 2
IA	Incomptine A
HKII	Hexokinase II
VDAC1	Voltage-dependent anion channel 1
MOM	Mitochondrial outer membrane
ATP	Adenosine triphosphate
Cyt c	Cytochrome c
DOXO	Doxorubicin
UT	Untreated
CC_50_	Cytotoxic concentration 50
ED_50_	Effective dose of 50
LD_50_	Lethal dose 50
TI	Therapeutic index
ALDOA	Aldolase A
LDH	Lactate dehydrogenase
Bcl-2	B-cell lymphoma 2
